# The global status of research in breast cancer liver metastasis: a bibliometric and visualized analysis

**DOI:** 10.1080/21655979.2021.2006552

**Published:** 2021-12-11

**Authors:** Yanlong Shi, Wei Wei, Li Li, Qian Wei, Fei Jiang, Guozhi Xia, Hongzhu Yu

**Affiliations:** a Department of General Surgery, Fuyang Hospital Affiliated to Anhui Medical University, Fuyang, P.R. China; b School of Nursing, Anhui Medical University, HeFei, P.R. China

**Keywords:** Bibliometrics, liver metastasis, breast cancer, prognosis

## Abstract

This study aimed to investigate the distribution laws and research frontiers of international literature, so as to present a holistic bibliometric evaluation of the studies on breast cancer liver metastasis(BCLM). Data were collected from the Web of Science Core Collection database, including publications, year, country, journal, author and keywords. The software VOSviewer and CiteSpace were used for bibliometric coupling, co-authorship, co-citation and co-occurrence analysis. In total, 1,031 publications were analyzed from 2004 to 2020 on BCLM. The year with the highest number of publications was 2006, with 103 papers. The United States, followed by China and Germany were the leading countries on BCLM, accounting for 59% of the whole. The journals that published about BCLM were mainly located in Q1/Q2. Keywords co-occurrence analysis divides BCLM into five clusters:‘basic research’, ‘auxiliary diagnosis and therapy’, ‘liver resection’, ‘clinical trial’ and ‘prognosis’. Main treatment therapies were the latest focus. Burst detection indicated that the trends in BCLM concentrated on subtype and SEER. There is apparently brighter perspective for BCLM research in the coming years, especially in liver resection, subtype and bioinformatics. The consequence of our study as the exclusive scientific evaluation offered an integral overview of BCLM, particularly for research focus and future directions, which can further accurately guide scholars on diagnosis, treatment, and personalized prevention.

## Background

Breast cancer(BC) is the most common malignancy in women worldwide, which accounting for approximately 30% of new case [1]. With advanced in diagnostic techniques and therapeutic methods, the clinical prognosis of BC patients has prominently improved over the years, with the 5-year survival rate exceeding in 90% in early BC [2]. However, what the leading cause of treatment failure and mortality in patients remains to be distant metastasis. And it is considerable that nearly 2%-6% of patients have already developed metastases at first diagnosis [3]. The major metastatic sites are bone(85%), liver(40–50%), pleura or lung(20%) and brain(6%-16%) [4]. Particularly, compared with bone and lung metastases, patients with liver metastases are associated with a worse outcome, with median survival live up to only 14–16 months, no matter of treatment [5]. On presentation, breast cancer liver metastasis(BCLM) may occur to abdominal discomfort, nausea and anorexia, even to the symptoms and physical signs suggestive of cachexia such as painful hepatomegaly, jaundice and ascites [6].

At present, the research related to BCLM has gradually emerged a certain priority, but there are still some inconsistencies in relevant conclusions, let along the lack of studies to tease out the preexisting literature from a quantitative perspective [7,8]. Although some scholars have adopted meta-analysis to discuss the topics with paradoxical conclusions, the profundity of discussion is relatively limited [9,10]. Simultaneously, it’s inescapable that the results by discussion is affected by the subjectivity of researchers. In addition, many scholars’ study on BCLM is generally restricted to the comprehensive reading of documents and the summary of personal clinical experience, lacking the necessary integrity and macroscopic [11]. Bibliometrics is a new approach for statistics of research outputs, which provides the qualitative and quantitative characteristics of literature for investigators by analyzing the measurement indicators including country, journal, institution and keywords, so as to describe the current trend and uncover the frontiers in a field [12–14]. It has testified important to various biomedical domains, containing inflammation, genetics and cancer [15–17]. What’s more, Bibliometric analysis has made great contributions to the formation of disease treatment and clinical guidelines [18]. However, there is no study evaluated scientific outputs in the BCLM field yet. Thus, it is very necessary to probe into the current state of research on BCLM.

In view of this, we systematically investigated scientific outputs related to BCLM from 2004 to 2020, so as to reveal the current research trend of BCLM. We then extensively explored the collaboration between authors, institutions, and between countries. Furthermore, we comprehensively analyzed the keywords, cited-references to identify its research focus and core hotspots, with a view to offer news ideas for clinical diagnosis and treatment of BCLM in the future.

## Material and methods

### Data source and retrieval strategies

**Figure 1.**
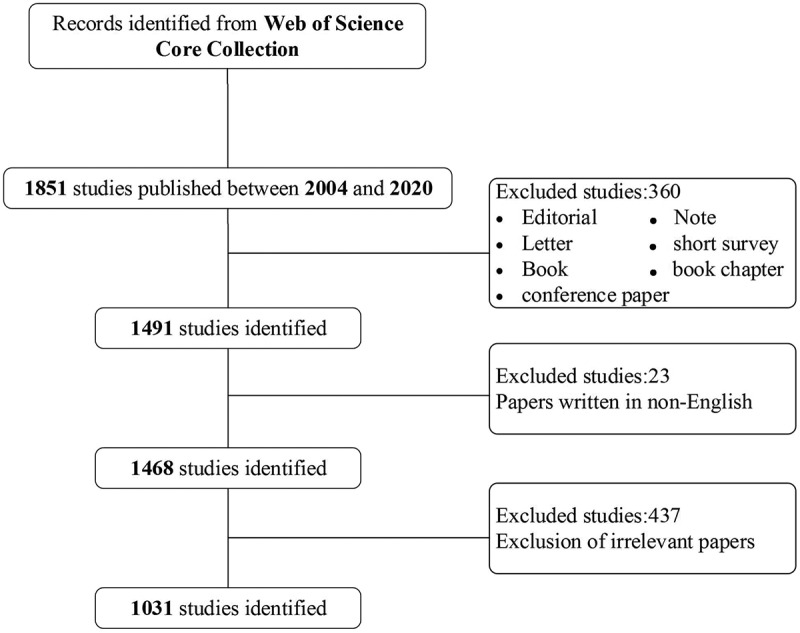
Flow diagram of screening process related to BCLM research

All publications related to BCLM came from Web of Science Core Collection (WOSCC) until 31 December 2020, which was known to harbor relatively dependable database, covering more than 12,000 of the highest impact, quality scientific journals [19]. The detailed data retrieval strategies and inclusion procedure of this study are shown in Figure 1. The search strategies were as follows: TS = ((‘breast cancer’ OR ‘breast carcinoma’) AND (‘liver metastas*’ OR ‘hepatic metastas*’)) OR ‘breast cancer liver metastas*’ OR BCLM OR ‘Liver metastas* from breast cancer’ OR LMBC. Besides, since this study didn’t include any animal or experiments, ethical consent was not required. Three authors(Yanlong Shi, Qian Wei and Wei Wei) searched and screened independently. If there was a difference between them, discussed it or turned to the fourth author(Hongzhu Yu) for help.

### Data collection

The bibliographic information were downloaded from the WOSCC database, including title, authors, year of publications, country/region, institution, keywords, citations, abstract and reference. Documents was downloaded in plain text format. The impact factor(IF) was derived from the Journal Citation Reports (JCR) 2019. VOSviewer 1.6.13 software was used to extract all data eligible for inclusion in this study by two investigators alone, and then imported into Microsoft Excel 2019 and Citespace V (Drexel University, Philadelphia, PA, USA). Where there was any conflict, the third investigator resolved it.

### Bibliometric indicators and visualization

Microsoft Excel (version.2019; Microsoft Corporation; Redmond, Washington) was conducted to analyze and draw the leading journal messages, such as annual publication, total number of citations and impact factor et al. And we summarized the relevant information of crucial co-cited references. In addition, we used Excel to make the world distribution map of the number of articles issued by countries.

VOSviewer (The Center for Science and Technology Studies; Netherlands) was used to analyze bibliographic coupling indicators and cooperation among countries/regions, institutions and authors, and then the maps were presented through network and overlay visualization [20]. Moreover, the high-frequency co-cited references and keywords were clustered by VOSviewer. A node represented one reference, and the size of the node was positively correlated with the frequency of co-citation of references. The larger the node, the more publications. The lines between nodes represented the strength of the connection. Different colors expressed different clusters in the network visualization [21].

Keywords timeline and bursts detection were implemented by CiteSpace V [22]. Afterward, we structured a timeline view of co-cited reference and a dual-map overlay of journal interrelated to BCLM. These indicators uncovered hotspots and frontiers, which were reflected the future research focus and trends to some extent. The parameters were as follows: time span (2004–2020), year of slice (1), selection criteria g-index(k = 25), link retaining factor (LRF = 3), look back years (LBY = 8), e = 2.0, pruning (Pathfinder) [23].

## Results and discussion

Bibliometrics was widely used to process quantitative and visual analysis scientific outputs in biomedical field, which not merely provided the basis for the diagnosis and treatment of diseases, but also forecast the direction of future of development. In this trial, we systematically investigated and summarized the distribution characteristics and research frontiers related to BCLM from 2004 to 2020 to reveal the current research trend of BCLM. A growing number of publications have been emerged since the 21st century, especially in the United States and China. The collaboration analysis demonstrated that there was limited collaboration between authors, institutions and countries. Moreover the prognosis defined by subtypes and bioinformatics is currently hotspots and highlights. Our study offered an integral overview of BCLM, particularly for research focus and future directions, which can further accurately guide scholars on diagnosis, treatment, and personalized prevention.

### General distribution characteristics analysis

#### Analysis of distribution of publications and citations

**Figure 2.**
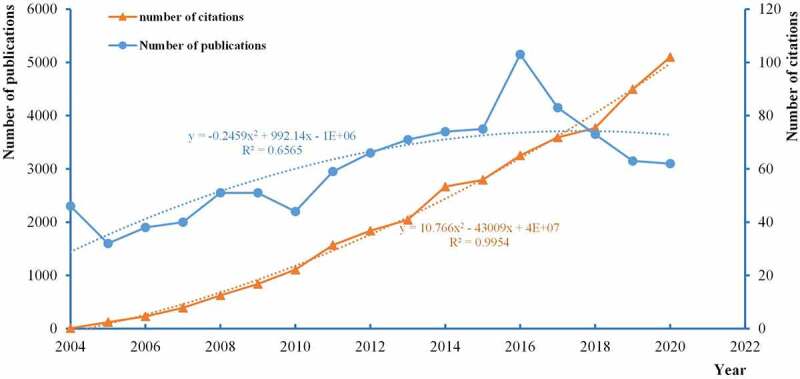
The annual number of publications and citations on BCLM from 2004 to 2020

Based on manual screening with patience, there were 1031 publications interrelated to BCLM from 2004 to 2020. As illustrated in Figure 2, the research plotted the number of publications and citations per year, which carried out mathematical function fitting for the curves of annual number of volume and times cited. Specifically, the quantity of articles had increased since 46 in 2004, culminating the peak in 2016 with a total of 103, but decreased thereafter. But it’s not said that the development of BCLM have encountered bottleneck or have no longer a hotspot. And the fluctuation of the number of documents each year demonstrated the relevant BCLM research was immature and deserved to be explored and improved. Besides, in terms of curve fitting, the trend will be level off. In addition, as time goes on, the number of citations grew exponentially, indicating that research on BCLM under the spotlight. To sum up, we estimated that there was apparently brighter perspective for BCLM research in the coming years.

#### Analysis of distribution of countries/regions

**Figure 3.**
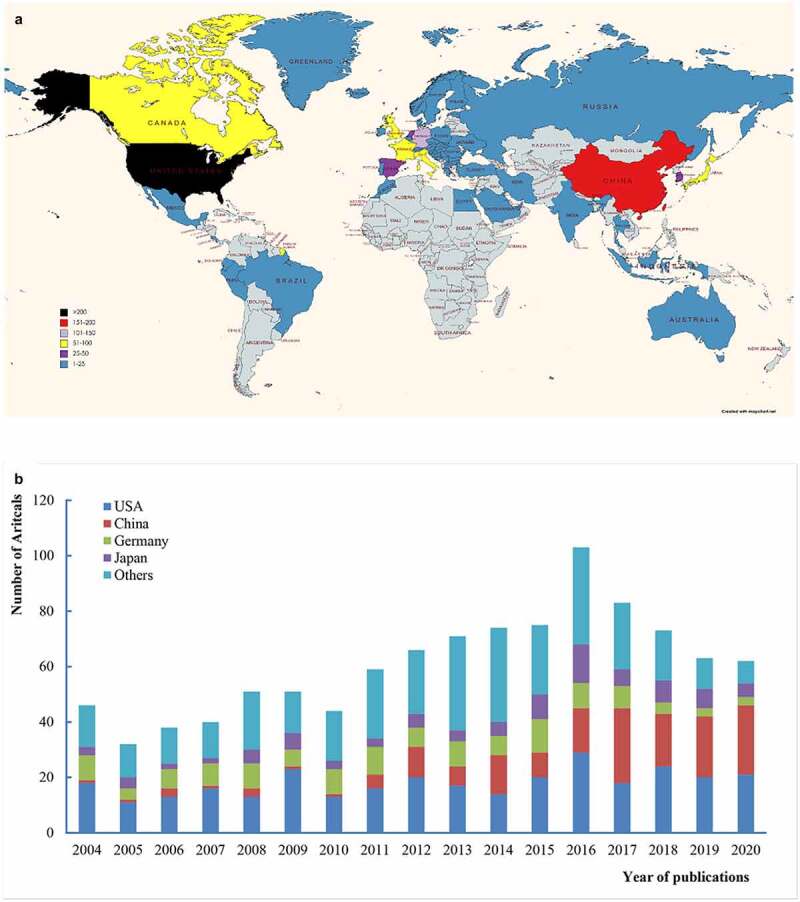
(a) Global map of countriesregions contributing to BCLM. (b) The distribution trend of the top 4 countriesregions by year

The world map indicated scientific output of countries with contribution to BCLM research. As the map of the world shown, among all 55 countries/regions from which publications on BCLM originated, the United States, followed by China and Germany were the leading countries in this field, with 605 articles came out, accounting for 59% of the whole(Figure 3(a)). Of note, the most of top productivity countries belong to developed countries but China and South Korea. This phenomenon reveals that the number of articles is intimately bound with economic development.

A bit farther on are Figure 3(b), we compared the trends in the number of articles between top four countries and others by year. It has been noticed that the United States is always the most energetic country in this domain. Chiefly, as time advance, The number of publications in China has been increasing continuously over past years, which reflects the national places a high value on BCLM research. However, despite of Germany ranking in 4, the key role to entire world is fading away in this field.

#### Analysis of distribution of journals

**Figure 4.**
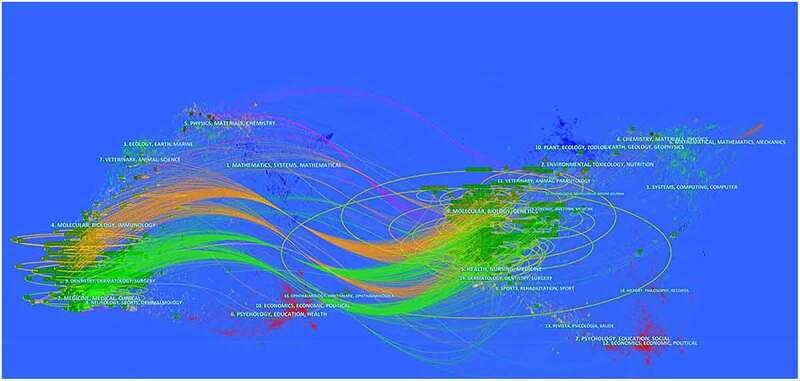
The dual-map overlay of journals related to BCLM

A dual-map overlay atlas can be used for analysis of disciplines and journals, for the purpose of revealing the distribution of citing journals and cited journals concretely. In the Figure 4, the left geographical region represented the citing journal, and the right map was on behalf of the cited journal. Disciplines were distinguished by the color of lines. The citing journals related to BCLM research mainly pertained to surgery, medicine, medical, clinical, molecular, biology and immunology journals, whereas the cited references were primarily published in molecular, biology, genetics, health, nursing, medicine and forensic, anatomy journals. Therefore, BCLM is closely connected with basic and clinical subjects, and the construction of multidisciplinary management needs to be further strengthened in the future.

Generally, the journals are distributed discretely in the BCLM field. Table 1 ranks the top 10 most prolific BCLM-related journals. The journal with the first four related to BCLM was BMC Cancer, followed by Breast Cancer Research and Treatment, Annals of Surgical Oncology and the journal of Anticancer Research with a total of 394 articles. The two journals, the Clinical Cancer Research and Cancer Research, were the highest impact factors and citations, focusing principally on oncology. For example, the title ‘Lymphopenia as a Prognostic Factor for Overall Survival in Advanced Carcinomas, Sarcomas, and Lymphomas’, published by Cancer Research, mainly concluded that Lymphopenia was an independent prognostic factor for overall survival and progression-free survival of BCLM1. In terms of JCR 2019 standard, the first 10 journals were principally situated in Q1 partition. Furthermore, the journal with the highest impact factor were Clinical Cancer Research(10.107) and Cancer Research(9.727). These outcomes disclose that the majority of articles are published in authoritative journals, suggesting that BCLM is attracting the attention of most scientists around the world. Moreover, combined with the country of publications and top productivity journals, it’s not too difficult to detect the Unite States is in leading position, and BCLM requires more collaboration between authors, between institutions, between countries.

**Table 1.** The top 10 journals related to BCLM

Rank	Journal	Number	Country	Citation	Average	IF*	Quartile in
					citation		category
1	BMC Cancer	26	England	353	13.577	4.430	Q2
2	Breast Cancer Research And Treatment	25	United States	499	19.96	4.872	Q2
3	Annals Of Surgical Oncology	22	United States	782	35.545	5.344	Q1/Q2
4	Anticancer Research	22	Greece	222	10.091	2.480	Q4
5	Oncotarget	21	United States	489	23.286	5.168	Q1/Q2
6	Cancer Research	19	United States	2669	140.474	12.701	Q1
7	Clinical Cancer Research	18	United States	979	54.389	12.531	Q1
8	Plos One	18	United States	711	39.5	3.240	Q2
9	Breast	17	England	192	11.294	4.380	Q1/Q2
10	Journal Of Vascular And Interventional Radiology	17	United States	480	28.235	3.464	Q2

*The impact factors (IF) of journals were obtained from the 2020 Web of Science Journal Citation Reports (JCR).

### Co-authorship analysis

#### Co-authorship analysis of the excellent authors

**Figure 5.**
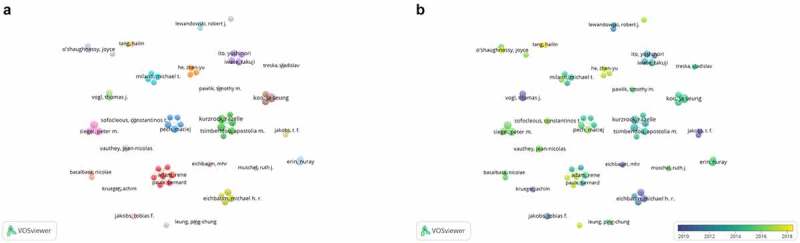
Co-authorship analysis of the excellent authors in the field of BCLM. (a) Network visualization map of the top 65 authors collaboration. (b) Overlay visualization map of the top 65 authors collaboration

A sum of 60 authors had generated more than 4 documents(Figure 5(a)). Except for co-operating authors of Naing Aung, followed by Adam Rene, and Kurzrock Razelle closely, other collaborative network of individual authors were scattered on BCLM, especially, Krueger Achim, Treska Valdislav and Meyer Carsten. From the perspective of cooperation relationship, this indicates that the current research is dominated by individuals, and no large academic community has been formed. Naing.A is the author who collaborates with other authors frequently, such as Kurzrock, R and Hong, DS. Their paper, titled ‘P53 Mutations in Advanced Cancers: Clinical Characteristics, Outcomes, and Correlation between Progression-Free Survival and Bevacizumab-Containing Therapy’ published by Oncotarget in 2013, discovered P53 Mutations were closely associated with livers metastasis, and Bevacizumab had shown promising results in patients with p53 mutated tumors [24]. This finding has been further studied in varieties of cancer, opening the door of the correlation between P53 mutations and tumor metastasis. Besides, Adam,R, Paule, B and Giacchetti, S et.al. Jointly found hepatic resection was a viable therapy option for patients with BCLM in 2006, which enlightening many scholars to delve into the treatment of BCLM by hepatectomy in depth [25]. Although the treatment of BCLM is still controversial, most clinician prefer hepatic section currently [26,27].

According to the overlay visualization map(Figure 5(b)), Tang, HL is the author who is most interested in BCLM recently, mainly prognostic factors of different modes of metastasis in breast cancer using population-based study exploring [28]. And it’s reported that patients with HBsAg(+) had a higher incidence of liver metastasis in breast cancer [29]. Moreover, the academic community consisting of Sun Jiayuan, He Zhenyu and Wang Sangang, et al., kept the research on BCLM in full swing.

#### Co-authorship analysis of the influential countries/regions

**Figure 6.**
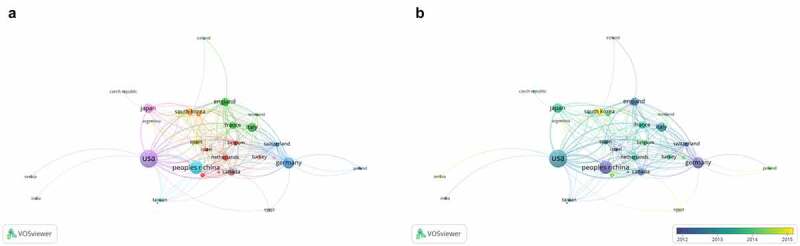
Co-authorship analysis of the influential countries/regions in the field of BCLM. (a) Network visualization map of collaborations among the first 32 countries/regions. (b) Overlay visualization map of of collaborations among the first 32 countries/regions

A total of 31 countries that published more than 5 documents were identified by VOSviewer. As illustrated in Figure 6(a), the Unite States has a strong working relationship to Japan, Germany and China. On the basis of our study, the Unite States was the most prolific contributor and the most extensive collaborator. To our surprise, the Unite States as well as other countries, such Sweden, Germany and Spain collectively published a paper on the journal of Nature, ‘Tumour exosome integrins determine organotropic metastasis’, which became a continually cited and hottest article with a total of 1796 times, and provided a new direction for tumor metastasis called exosomal integrins [30]. Interestingly, although the publications of Germany(124) is less than China(178), its total link strength is exceed in China, 40 and 36 respectively. For example, the article entitled ‘Identification of a population of blood circulating tumor cells from breast cancer patients that initiates metastasis in a xenograft assay’ written by Baccelli, I,Schneeweiss, A and et.al. that published in Nature Biotechnology with cited 631 times [31]. This is the first time that the existence and phenotype of metastasis-initiating cell(MICs) in circulating tumor cells(CTCs) have been verified in a xenografts experiment. And MICs may provide a turning point in the diagnosis and treatment of metastatic breast cancer. From which we can learn, cooperation between countries is a powerful catalyst for promoting knowledge renewal and practical change.

Furthermore, on the overlay visualization map(Figure 6(b)), it’s noticed that the trend of cooperation between countries has been declining in recent years. This condition is prone to cause regional development imbalance, so each notion should be attempt to ascertain primary causes for this phenomenon.

#### Co-authorship analysis of the active institutions

**Figure 7.**
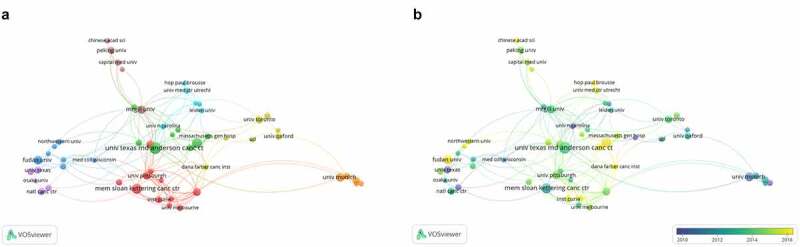
Co-authorship analysis of the active institutions in the field of BCLM. (a) Network visualization map of the leading 60 institutions. (b) Overlay visualization map of the leading 60 institutions

As shown in Figure 7(a), we produced a collaborative network visualization for institutions with a minimum of 6 articles. When analyzing the characteristic of the institution collaboration network, we detect there display regional concentration, as well as overall dispersion. The University of California Francis was among the most zealous institutions, followed by UTMD Anderson Cancer Center and University of pittsburgh, forming an influenced coo perative troop. Surprisingly, the number of publication of University of California Francis was 14, but the strength links between institutions was highly than other institutions. Their latest research suggested that Atezolizumab plus nab-paclitaxel can be used as first-line treatment for metastatic triple-negative breast cancer [32]. Moreover, vessel co-option may be related to resistance to anti-angiogenic studied by UTMD Anderson Cancer Center and McGill University in 2016 [33]. These breakthroughs highlight the importance of institutional collaboration on BCLM research. However, though the publications of Memorial Sloan Kettering Cancer Center was listed in second in the field of BCLM, the total link strength was thin on the ground, with the sum of only 7.

The distribution of institutions in varying time periods were presented in Figure 7(b)). As you look closely, University of carolina, University of Munich as well as Massachusetts General Hospital was the main constitutions who conducted researches before 2011 in this area. Then, Dana-Farber Cancer Institute, followed by Sun Yat Sen University and Shandong University were one of the hottest institutions after 2015, major direction containing tumor metastasis [34], hepatic resection and survival outcomes [35]. Particularly, Dana-Farber Cancer Institute et al. discovered that weaning-induced liver involution, a newly unrecognized biology, possibly was the cause of poor prognosis in postpartum breast cancer patients [36]. In short, we should actively organize academic conferences to promote the innovation and development of BCLM in the world.

### Ca-citation and co-occurrence analysis

#### Ca-citation analysis of co-cited references

**Figure 8.**
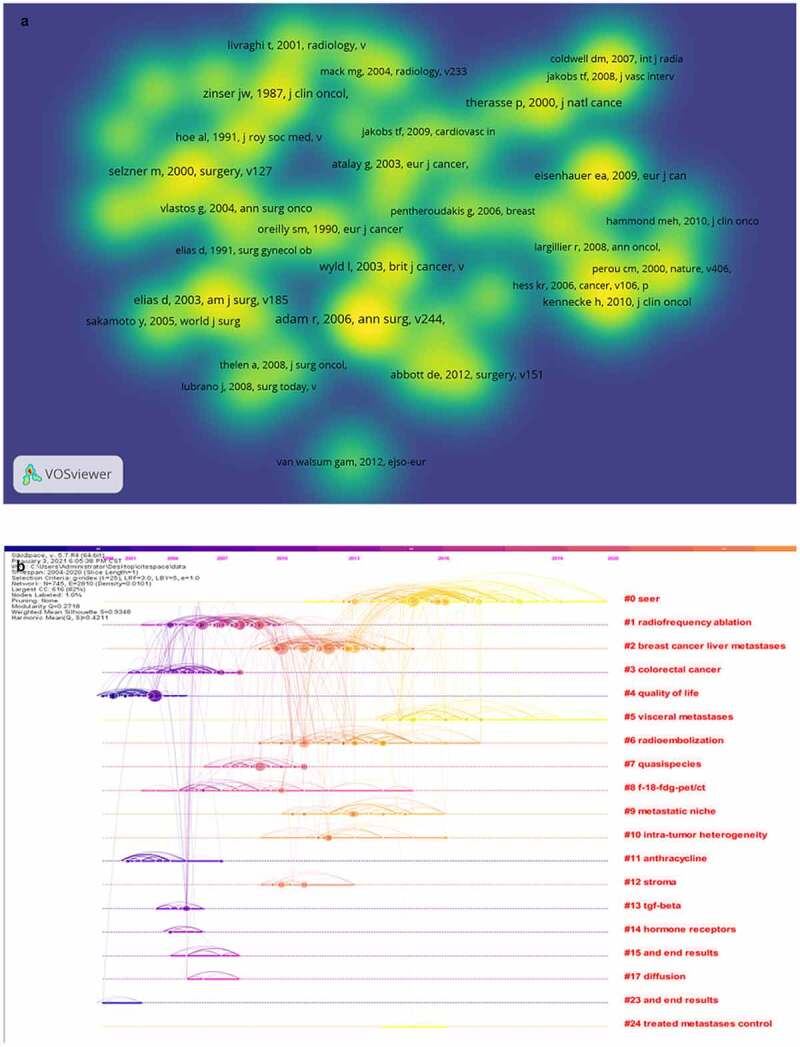
A. Ca-citation analysis of co-cited references in the field of BCLM. (a) Density visualization map of the top 52 co-cited references. (b) Time line and clustering view of all of co-cited references

Co-cited references not only present the core literature that plays a pivotal role in the evolutionary process within a field, but also unmask the changing trends in research priorities [37]. Figure 8(a) presented the network visualization map of the 52 most co-cited references, which was equal or greater than 20 times. As can be seen from the picture, most of the co-cited references came from top journals, with the main disciplines involved being oncology and surgery. This demonstrates that BCLM has been taking seriously by many scientists. Subsequently, we listed the top of 10 co-cited references by title, corresponding authors, country, journal etc., to provide the structural characteristic of the region of BCLM and its evolution for scholars (Table 2). In terms of citations frequency, the maximum number of references was merely 75 times, published by Adam et al. in 2006, followed by Cl et al. and Elias et al. Otherwise, there were 5 different countries from the first 10 references, consisting of the Unite State, England, France, Canada and the Kingdom of Cambodia.

**Table 2.** The top 10 co-citation references related to BCLM

Rank	Title	Country	Corresponding author	Year	Journal	
1	Is Liver Resection Justified for Patients	France	Rene´ Adam	2006	Annals of Surgery	
	With Hepatic Metastases From Breast Cancer					
2	Liver metastases from breast cancer:	USA	Pierre-Alain Clavien	2000	Surgery	
	Long-term survival after curative resection					
3	An attempt to clarify indications for hepatectomy	France	Dominique Elias,	2003	The American Journal of Surgery	
	for liver metastases from breast cancer					
4	Prognostic factors for patients with	UK	Dr L Wyld	2003	British journal of cancer	
	hepatic metastases from breast cancer					
5	Clinical Course of Breast Cancer	USA	By Juan W. Zinser	1987	Journal of clinical oncology	
	Patients With Liver Metastases					
6	New Guidelines to Evaluate the Response to	Belgium	Patrick Therasse	2000	Journal of the National Cancer Institute	
	Treatment in Solid Tumors					
7	Metastatic Behavior of Breast Cancer Subtypes	USA	Hagen Kennecke	2010	JOURNAL OF CLINICAL ONCOLOGY	
8	Breast liver metastases–incidence, diagnosis and outcome	UK	I Taylor	1991	Journal of the Royal Society of Medicine	
9	New response evaluation criteria in solid tumors:	Canada	E.A. Eisenhauer	2009	EUROPEAN JOURNAL OF CANCER	
	Revised RECIST guideline					
10	Long-term Survival After An Aggressive Surgical Approach in Patients With Breast Cancer Hepatic Metastases	USA	Jean-Nicolas Vauthey	2004	Annals of Surgical Oncology

In addition, we constructed a timeline map to display the vital clusters of co-cited references(Figure 8(b)). In terms of time distribution, clustering is relatively concentrated, mainly including #0 seer, #1 radiofrequency ablation, #2 breast cancer liver metastases, #3 colorectal cancer, #4 quality of life and #5 visceral metastases and metastatic niche. It’s obvious that #0 cluster(seer) and #5 cluster(visceral metastases) chiefly took place between 2015–2019, which indicated that these clusters were the most popular direction in BCLM research.

#### Co-occurrence analysis of keywords

**Figure 9.**
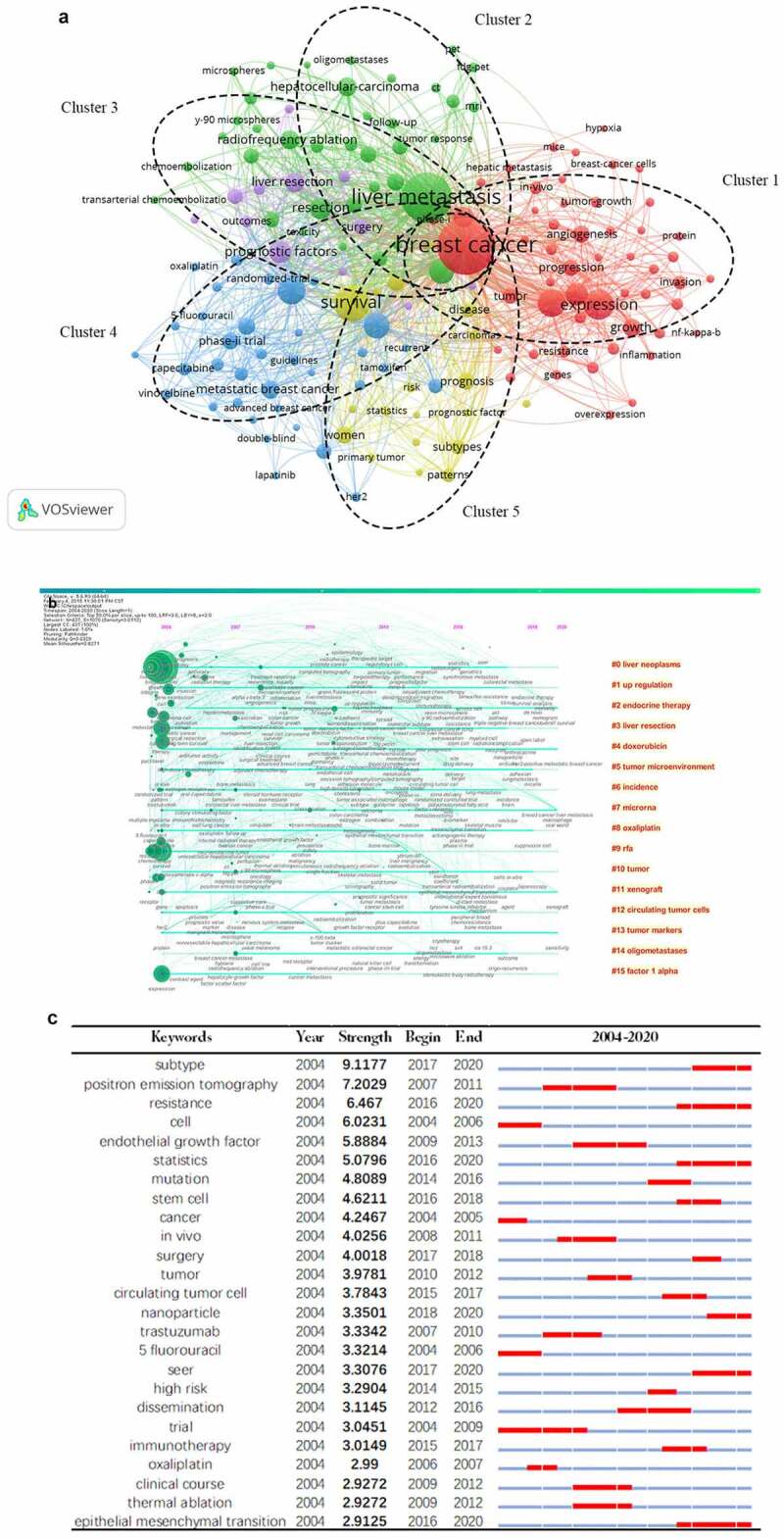
Co-occurrence analysis of high frequency keywords in the field of BCLM. (a) Network visualization map of co-occurrence and clustering analysis of the frequent 156 keywords. (b) Timeline and clustering view of keywords for BLCM. (c) Top 25 keywords with the strongest citation bursts of BCLM

The co-occurrence keywords network visualization map with extraction frequency of 10 times or more was shown in Figure 9(a). There are 156 high frequency keywords extracted from 1031 articles, and can be classified into five categories, as follows: cluster1:‘basic research’(red color) including angiogenesis, expression, progression and in vivo; cluster2: ‘auxiliary diagnosis and therapy’(green color) including radiofrequency ablation, mir, pet and microspheres; cluster3(purple color): ‘liver resection’ including surgery, liver resection, outcomes and prognostic factors; cluter4:‘clinical trial’(blue color) including therapy, phase-ii trial, randomized trial, capecitabine and chemotherapy; cluster5:‘prognosis’(yellow color) including survival, subtypes, statistics and risk.

The timeline map of keywords can clearly observe the time span of each cluster and the development trend of a specific cluster, explore the time characteristics of research field reflected by each cluster, and thereby verdict the evolution trend of hotspots. According to the Figure 9(b), the longest lasting heat was Endocrine therapy, Doxorubicin, Microrna, Xenograft and Tumor markers, starting in 2004 and continuing since. These latest keywords are Survival analysis,Open label, Radiation enlargement and Laparoscopy. And the shortest duration cluster was RFA and Factor 1 alpha. Liver neoplasms were the most intersecting line, illustrating its frequent interconnection with other directions in breast cancer.

Burst detection can uncover theoretical trends and new topics that are emerging or emergent in a field. The top 25 keywords with the strongest citation bursts are presented in Figure 9(c). From 2004 to 2010, ‘trial’, ”oxaliplatin” and ”trastuzumab” were the focus of research, indicating that the research of BCLM converged on clinical trials during this period. Then, it was transformed into ‘endothelial growth factor’, ‘mutation’, ‘immunotherapy’ and ‘circulating tumor cell’ continued 2011 to 2017. Recently, the interests in this domain primarily convert to big data technology, including seer and statistics. Furthermore, the keyword, ‘subtypes’, possessed the highest burst strength, with the value of 9.118.

#### Research focus and future directions on BCLM

According to the ca-citation and co-occurrence analysis, we identified main treatment therapies, subtype and bioinformatics as research topics in the field of BCLM.

#### Main treatment therapies

There are two main types of therapy for BCLM: systemic treatment and local treatment. If untreated, the survival time of patients with BCLM is only 4–8 months. Furthermore, effective treatment can improve the prognosis of patients and prolong the survival appropriately [38].

At present, there is no clinical evidence to select the optimal local treatment among surgery, stereoradiotherapy and intrahepatic chemotherapy. With the development of multidisciplinary management, a growing number of clinician inclined to perform liver resection for patients with BCLM. A multi-center study involving 1,452 patients for non-colorectal and non-endocrine liver metastases who underwent liver resection showed that the overall 5-year and 10-year survivals were 41% and 22%, respectively, for surgically treated [39]. Furthermore, the median overall survival after hepatectomy was 41 months in highly selective patients with BCLM [40]. Although it has been suggested that surgery doesn’t confer a survival advantage over patients who receive systemic therapy alone, the significantly improved recurrence free period can provide sufficient chemotherapy time for patients [7]. Besides, Ruiz et al. investigated the survival of repeated hepatectomy in patients with recurrent BCLM [41]. The 5-year survival was 50% for repeat hepatectomy, higher than for patients who underwent only one hepatectomy. Importantly, a latest study found that The longer interval between surgery for breast cancer and the diagnosis of liver metastasis in patients with partial hepatectomy, the better the prognosis [27]. However, the majority of cases were small sample retrospective studies, mainly involving isolated patients with BCLM. In the future, it is necessary to carry out prospective randomized controlled studies to obtain higher-level clinical evidence, which is of great significance for clarifying hepatectomy.

Systemic therapy of BCLM depends on estrogen receptor (ER), progesterone receptor (PR), and human epidermal growth factor receptor 2 status (HER2). Anthracyclines or taxanes are generally preferred chemotherapy regimens. A retrospective study of patients who received anthracycline or anthracycline plus taxane based chemotherapy, 66.4% of the patients achieved objective response. Surprisingly, 12.1% of patients survived for more than 60 months [42]. However, it is more difficult to control the progression of BCLM with chemotherapy alone [43]. As a result, other methods combined with chemotherapy have gradually drawn increasing attention from scientists, such hepatic arterial therapy and regional inductive moderate hyperthermia [44–46].

Hepatic arterial embolism(TAE), transcatheter hepatic arterial chemoembolization (TACE) and hepatic arterial infusion chemotherapy(HAI) may be a safe a safe alternative way for BCLM patients, with the main advantage of blocking the tumor blood supply while greatly increasing the local drug concentration.

Although radiofrequency ablation(RFA) has been widely used in patients with primary liver cancer and colorectal cancer liver metastasis, RFA for BCLM remains controversial [47,48].The progression-free survival to sum up was 45 months, with 1-, 2-, and 3-year survial rates of patients with BCLM treated with RFA were 87%, 68%, and 48% [49]. Meloni et al performed RFA on 87 BCLM lesions with an initial complete inactivation rate of 95% [50].Moreover, in the local treatment of BCLM, RFA is a useful adjuvant for systemic chemotherapy [51].Yunus et al. reported that laparoscopic RFA plus systemic therapy had an unexpected effect than systemic therapy alone for patients with multiple intrahepatic metastases or with extrahepatic metastases [52]. Particularly, the size of liver metastases affects therapeutic effects of RFA. For example, patients with tumor diameter≥2.5 cm had a worse prognosis than those with tumor diameter<2.5 cm [50]. In addition, Andrea et al. showed that the complete ablation rate of BCLM < 30 mm and >30 mm was 81% and 43%, respectively [53].

Radioembolisation with yttrium-90 has been raising hopes for patients with therapy-refractory and unresectable BCLM, especially in a low tumor burden or sequential lobar treatment [54]. However, elevated baseline bilirubin levels and Eastern Cooperative Oncology Group (ECOG) status were associated with adverse outcomes [55]. Cianni et al. conducted radioembolisation with yttrium-90 on 52 eligible BCLM patients. Based on Response Evaluation Criteria in Solid Tumor (RECIST), the partial response and stable disease were seen in 56% and 35% of patients respectively [56].The latest systematic review included 452 patients from 12 cohort studies. Tumor control rate reached 81% at 6–15.7 months follow-up. And the overall survival after radioembolization was 3.6–20.9 months [57]. Surprisingly, Bernard et al. reported that mean tumor dose >70 Gy was of great significance to tumor response and prolonging OS in patients [58]. Radioembolisation with yttrium-90 may become an effective approach, however, it will urgently need to be proved in randomized trials in the future.

Recently, bioinformatics has become an increasingly essential area of medical field, which mainly investigates both genomics and proteomics. Gene expression profile, the most common form of bioinformatics, is to quantitatively and qualitatively analyze mRNA expression in specific tissues or cells by DNA sequencing, so as to reveal relevant biological mechanisms and provide potential ideas for the treatment and prediction of tumors [59]. For example, by analyzing somatic changes in 617 cases of metastatic breast cancer, Bertucci et al. identified 9 driver genes with higher mutation frequency compared to early-stage breast cancer [60]. This discovery will provide more standardized guidance for clinical trials. Lin et al. reported that hsa circ 21,439 and hsa circ 11,783 may associated with diagnosis and therapy targets of BCLM [61]. In addition, nomogram, as a novel frontier that has risen recently, is a visual prognostic model based on Surveillance, Epidemiology, and End Results (SEER) database, which can intuitively reflect the interrelationship between each variable, as well as facilitate physicians to quickly determine the prognosis of patients [62]. Lin et al. constructed a trustworthy implement to discern and forecast BCLM by utilizing breast cancer clinicopathological variables and distant metastasis status [63]. The prognosis model developed by Zhao et al. can not only foretold the survival of patients with de novo metastatic breast cancer responsibly, but also guided whether surgery was indicated in the clinic [64]. Surprisingly, Linjie et al created a tool to determine the prognosis of patients with BCLM, which found and validated molecular subtype, metastatic interval, extrahepatic metastases and liver function status as independent prognostic factors [65]. It is not difficult to discover that the establishment of prognosis model can not merely evaluate the risk of each prognostic factor, but also further optimize individual treatment. Notably, whether these prognostic models based population study in BCLM can be dependable still need to be further validated. In general, in the age of big data, we should attach much important to the law of diseases occurrence through the analysis of statistical data.

The focus of subtype research has converted from treatment in 2012 to prognosis after 2017, which is consistent with the keyword ‘cluster 5’ [66,67]. With respect to subtype, this has been a frontier field in the research of BCLM since 2017. As we know, various subtypes of BCLM are closely associated with treatment and prognosis. And the common subtypes are luminal B and HER2-positive, with a poor prognosis [28]. CHEN et al. also reported that the median survival of triple-negative breast cancer (TNBC) patients with liver metastasis is only 15 month, which is closely related to the advanced stage of tumor, increased risk of brain and the increased risk of mortality [68]. Furthermore, Chun et al. analyzed the prognostic impact of various subtypes in patients with BCLM who underwent hepatic resection, with median OS surpassing 75 months for HER2-positive and luminal B. What makes it terrible was that 76% of patients relapsed had recurrence or extrahepatic diseases after resection. Interesting, a number of studies on subtype prognosis were based on SEER [69]. In the future, it is necessary to further study the subtypes of BCLM, which is helpful for further early diagnosis and treatment of patients.

## Strengths and limitations

This study was the first to systematically and comprehensively illustrate the characteristics and trends of scientific literature on BCLM. However, to be honest, several limitations still need to be noted. Firstly, we only chose to publish articles in English, which may lead to selection bias. Secondly, although the majority of publications included in the WOSCC database are of high quality, it will inevitably result in bibliography omissions. Finally, some newly published high-quality papers may be sightly lower than classic papers temporarily.

## Conclusion

This study has analyzed systematically and summarized the distribution characteristics and research frontiers by bibliometrics concerning BCLM. Over the last decade or so, a crowd of countries with strong scientific creativity like the United States has emerged, such as China, Greece and Japan. More collaborations among authors, institutions, and countries are needed to probe into the treatment of BCLM, particularly for liver resection and RFA, through the use of randomized controlled trial. The founding also obtained that latest frontiers in BCLM was to define the prognosis by analyzing subtypes and bioinformatics.

## Highlights

- Bibliometrics provides novel insights and directions for the diagnosis and treatment of BCLM.
- What attach much importance to explore the study of surgery and prognosis.
- There is apparently brighter perspective in subtype and screening biomarkers for BCLM.
